# Effects of different dietary energy and protein levels and sex on growth performance, carcass characteristics and meat quality of F1 Angus × Chinese Xiangxi yellow cattle

**DOI:** 10.1186/2049-1891-5-21

**Published:** 2014-04-16

**Authors:** Lingyan Li, Yuankui Zhu, Xianyou Wang, Yang He, Binghai Cao

**Affiliations:** 1 National Beef Cattle Industry and Technology System, College of Animal Science and Technology, China Agricultural University, Beijing 100193, China; 2 Tin Wah Industrial Co.Ltd, Shimen Industrial Area of Lian Yuan 417100, Hunan Province, China

**Keywords:** Carcass characteristics, Energy, F1 Angus × Chinese Xiangxi yellow cattle, Growth performance, Meat quality, Protein, Sex

## Abstract

**Background:**

The experiment evaluated the effect of nutrition levels and sex on the growth performance, carcass characteristics and meat quality of F1 Angus × Chinese Xiangxi yellow cattle.

**Methods:**

During the background period of 184 d,23 steers and 24 heifers were fed the same ration,then put into a 2 × 2 × 2 factorial arrangement under two levels of - dietary energy (TDN: 70/80% DM), protein (CP: 11.9/14.3% DM) and sex (S: male/female) during the finishing phase of 146 d. The treatments were - (1) high energy/low protein (HELP), (2) high energy/high protein (HEHP), (3) low energy/low protein (LELP) and (4) low energy/high protein (LEHP). Each treatment used 6 steers and 6 heifers, except for HELP- 5 steers and 6 heifers.

**Results:**

Growth rate and final carcass weight were unaffected by dietary energy and protein levels or by sex. Compared with the LE diet group, the HE group had significantly lower dry matter intake (DMI, 6.76 vs. 7.48 kg DM/d), greater chest girth increments (46.1 vs. 36.8 cm), higher carcass fat (19.9 vs.16.3%) and intramuscular fat content (29.9 vs. 22.8% DM). The HE group also had improved yields of top and medium top grade commercial meat cuts (39.9 vs.36.5%). The dressing percentage was higher for the HP group than the LP group (53.4 vs. 54.9%). Steers had a greater length increment (9.0 vs. 8.3 cm), but lower carcass fat content (16.8 vs. 19.4%) than heifers. The meat quality traits (shear force value, drip loss, cooking loss and water holding capacity) were not affected by treatments or sex, averaging 3.14 kg, 2.5, 31.5 and 52.9%, respectively. The nutritive profiles (both fatty and amino acid composition) were not influenced by the energy or protein levels or by sex.

**Conclusions:**

The dietary energy and protein levels and sex significantly influenced the carcass characteristics and chemical composition of meat but not thegrowth performance, meat quality traits and nutritive profiles.

## Background

Angus is one of the most popular breeds of cattle used in beef production because it has a considerable growth rate, high carcass yield and well-marbled meat. Xiangxi yellow cattle area breed of Chinese indigenous yellow cattle that is bred in the northwest of Hunan Province. It was included in *the National Protection List of Livestock and Poultry Genetic Resources* of China in 2006 [1]. The breed is well-adapted to low-quality roughage and high temperature environments, its mature weight does not exceed 400 kg, its growth rate is under 0.5 kg/d and the dressing percentage and longissimus muscle (LM) area are 49.48% and 46.75 cm^2^, respectively [2]. In the past, the low level of agricultural mechanization has meant that the yellow cattle in China were only used as draft animals. However as a result of rapid economic development, the standard of living in many communities has increased, which has produced a higher demand for beef in terms of both quantity and quality. Therefore, to meet the current market demands, methods to increase beef quality and quantity have been introduced, such as crossing superior foreign breeds with native breeds and manipulating nutrition. Dietary nutrition has important roles in growth performance, carcass quality and meat quality traits [3-5]. Previous studies have assessed the effect of nutrition on the growth performance, carcass characteristics and meat quality of Angus [6] or Angus crossbred cattle, such as Angus × Holstein–Friesian, Angus × Gelbvieh and Angus × Limousin cattle [7-9]. However, little research has been performed on the crossbred progeny of Angus and Chinese yellow cattle. Therefore, the aim of this study was to assess the effects of different dietary energy and protein levels and sex on the growth performance, carcass characteristics and meat quality of F1 Angus × Chinese Xiangxi yellow cattle.

## Materials and methods

### Animals and management

Animal care and procedures were approved and conducted under established standards of the College of Animal Science & Technology, China Agricultural University.

Twenty-three (23) male and twenty-four (24) female weaning calves of F1 Angus × Chinese Xiangxi yellow cattle were selected and transferred from the breeding centre to the fattening farm of Hunan Tin Wah Industrial Co., Ltd. The calves were the F1 progeny of purebred Angus bulls bred to dams of purebred Chinese Xiangxi yellow cows. After arriving at the fattening farm, all male calves were castrated and dewormed. The cattle at an average age of 6.5 mon were weighed and fed the same ration as during the background period (184 d). The animals were then placed in a 2 × 2 × 2 factorial arrangement to study the effects of two levels of dietary energy (TDN (Total Digestible Nutrients): 70%, 80% DM (Dry Matter)) and protein (CP (Crude Protein): 11.9%, 14.3% DM) and sex (S: male, female) on the growth performance, carcass characteristics and meat quality during the finishing phase. The cattle were divided into four treatment groups based on age, body weight (BW) and growth rate during the background period and body size. The treatment groups were:

(1) high energy and low protein(HELP; TDN: 80% DM,CP: 11.9% DM),

(2) high energy and high protein(HEHP; TDN: 80% DM,CP: 14.3% DM),

(3) low energy and low protein(LELP; TDN: 70% DM,CP: 11.9% DM),

(4) low energy and high protein(LEHP; TDN: 70% DM,CP: 14.3% DM).

Six steers and 6 heifers were placed in each treatment group, except for the HELP group, which contained 5 steers and 6 heifers. At the start of the finishing phase, a 14-day adaptation period was used for transition between rations which consisted of mixing 1/3 of the finishing ration with the previous ration for 7 d, then adding 2/3 of the finishing ration for the following 7 d and finally switching to the entirely new ration. The cattle were not implanted with any steroid hormones and were fed for 146 d until slaughter. All of the cattle were held in eight sheltered pens at a stocking density of 5 m^2^ per animal during the background period and were tied up to feed during the finishing period. The animals were fed twice a day at 0700 h and 1700 h and allowed to drink water freely. The nutrition levels of different phases and treatment groups are shown in Table 1.

**Table 1:** Feed ingredients and nutrition levels for background period and different treatment groups during finishing

**Item**	**Background**	**Finishing**			
		**HE**		**LE**	
		**LP**	**HP**	**LP**	**HP**
**Ingredient, % DM**					
Hybrid penisetum	20.00	—	—	—	—
Rice straw	20.00	20.00	20.00	30.00	30.00
Ground corn	29.90	66.54	61.03	41.23	36.83
Soybean meal	19.18	10.46	15.97	3.70	7.86
Cottonseed meal	7.45	—	—	4.32	7.56
Wheat bran	—	—	—	18.00	15.00
Limestone	0.67	1.00	1.00	1.00	1.00
Sodium bicarbonate	1.00	—	—	—	—
Salt	1.20	1.20	1.20	1.05	1.05
Premix^1^	0.60	0.80	0.80	0.70	0.70
**Nutrient level, % DM**					
DM	74.19	86.31	86.45	86.69	86.85
CP	16.52	11.96	14.34	11.90	14.30
TDN	68.22	79.78	79.87	69.95	70.03
NDF	34.27	21.60	21.81	33.09	33.24
ADF	20.53	11.27	11.59	17.75	18.40
Ca	0.42	0.39	0.40	0.41	0.42
P	0.31	0.23	0.24	0.35	0.38

^1^Vitamin and mineral premix contained per kilogram DM: Vitamin A, 154,000 IU; Vitamin D, 38,500 IU; Vitamin E, 3,500 IU; Fe, 9.0 g; Zn, 7.0 g; Mn, 14.0 g; Cu, 1.0 g; I, 138.0 mg; Se 30.0 mg; Co, 60 mg; Monensin, 30 g/1,000 kg.

### Growth performance

The cattle were weighed at the beginning and end of the background and finishing phases. The dry matter intake was recorded every 2 wk to calculate the average DMI during the background and finishing phases. Samples of the diet and refusals (uneaten feed) were collected every month for analysis using the standard methods of AOAC(2000) for DM, CP, Ca and P [10]. NDF (Neutral Detergent Fiber) and ADF (Acid Detergent Fiber) were determined following a modification of the procedure of Van Soest et al. [11]. Body measurements, including withers height, body length, chest girth and shin circumference were taken at the beginning and end of the finishing phase. The first two measurements were recorded using calipers and the latter two were recorded using a metal measuring tape.

### Carcass characteristics

At the end of the trial, all of the cattle were slaughtered. The hot carcass weight (HCW) and cold carcass weight(CCW, hot carcass × 0.98) were recorded to calculate the dressing percentage and the carcass composition. At the same time, a sample meat cut(2 cm × 5 cm × 3 cm), free of external fat and connective tissue, was also taken between the 6^th^ and 7^th^ ribs of the LM (*Longissimus dorsi* muscle) from the left side of each carcass. The sample was weighed, hung by a nylon cordin a plastic bag at 4°C for 48 h, then dried on absorbent paper before reweighing to ascertain the drip loss percentage, which was calculated by (initial weight-final weight)/initial weight. The carcasses were then put into a chiller at 0–4°C and aged for 7 d. The ultimate pH of the LM (12–13^th^ rib) was measured on the left body side at 48 h post-mortem using a pH electrode probe (Testo 205, Testo AG, Lenzkirch, Germany), and the following carcass linear measurements were recorded: length of carcass, depth of chest, length of leg [12], maximum girth of leg, lean thickness (muscle and subcutaneous fat) of leg, rib (between the 5^th^ and 6^th^ rib) and loin (between the 3^rd^ and 4^th^ lumbar vertebrae). The carcass composition (bone, fat and meat) was assessed by dissection of the 8^th^ rib, cut on the 8^th^ day post-mortem [13]. The LM area and fat thickness were measured between the 12^th^ and 13^th^ ribs of the LM using a plastic grid and Vernier caliper. Commercial meat cuts were dissected and named following the standard method by Chen [14] and were weighed after trimming. Based on the most popular and economic meat cuts in Chinese markets, the highrib, ribeye, striploin and tenderloin were considered as the top grade cuts, and the chunk tender, topside, outside flat, eye round, rump and knuckle were considered as the medium top grade cuts. The top and medium top grade cut yields were then calculated.

### Meat quality

The meat quality was determined from the sample (6.0 cm thick) removed from the longissimus muscle between the 12^th^ and 13^th^ ribs on the left sideof the body after carcass dissection, with no external fat or connective tissue. The meat samples were then frozen (-24°C) and transported to the China Agriculture University until analysis could be conducted. The meat samples were cut into three steaks using a saw before thawing. The first sample (2.54 cm thick) was used for calculating cooking loss by measuring the difference in weight before and after a period of heating to an internal sample temperature of 70°C in a 75°C water bath six 1.27-cm cores parallel to the muscle fiber orientation were then removed from the cooked sample for the instrumental measurement of tenderness by a texture analyzer (TA.XT plus, SMS, Godalming, Surrey, UK). The second sample (1.5 g) was allowed to thaw for 12 h at 1–2°C and then its water holding capacity (WHC) was measured by holding the sample under pressure (35 kg) for 5 min by a texture analyzer (TA.XT plus) fitted with a compression platen (diameter 7.5 cm) and reweighing. The following equation was used to calculate WHC.

$$X=\frac{M_{1}A‒\left(M_{1}‒M_{2}\right)}{M_{1}A}$$

Where X =% WHC, M_1_ = weight of sample before compression (g), M_2_ = weight of sample after compression (g) and A = total water content in the sample (%). The third sample was freeze-dried and then DM, crude protein, intramuscular fat, fatty acids (FA) and amino acids (AA) were measured.

The one-step extractive methylation procedure for fatty acids gain [15] was performed in a gas chromatograph (GC-2014, Shimadzu, Kyoto, Japan) with a capillary column (HP-88 100 m long, 0.25 mm diameter, 0.20 μm film. Agilent Santa Clara, California, America) using margaric acid (C17:0) as an internal standard. The oven temperature was programmed to provide three consecutive ramps, the first had an initial temperature of 120°C maintained for 1 min then increasedby 10°C/min until it reached 175°C,where it was maintained for 10 min; the second increased by 5°C/min until it reached 210°C, where it was maintained for 5 min and the third ramp increased by 5°C/min to 230°C, where it was maintained for 5 min. The carrier gas was helium at a flow rate of 2 mL/min. An automatic split/splitless injector with a 1/50 split and a temperature of 250°C was used. The injection volume was 1 μL. A flame ionization detector (FID) was used with an air flow of 450 mL/min, hydrogen flow of 40 mL/min and a detector temperature of 280°C. Fatty acids were expressed in gravimetric concentrations (mg/g of freeze dried sample).

Amino acids were determined in the dried, fat-free meat samples using a Shimadzu (10A VP DAD) high-performance liquid chromatograph (HPLC) following the procedure described by Wu [16].

### Statistical analysis

There were three major factors in the experimental design: dietary energy, protein and sex with each factor having two levels: energy (TDN: 70%, 80% DM), protein (CP: 11.9%, 14.3% DM) and sex (S: male, female). Therefore the effects of dietary energy, protein and sex and their interactions with growth performance, carcass characteristics and meat quality traits were analyzed using a 2 × 2 × 2 factorial arrangement (energy × protein × sex) using the GLM procedure of SAS (version 9.0, SAS Inst. Inc., Cary, NC, USA). When a significant effect of treatment was detected (*P* < 0.05), differences between the means were tested using Tukey’s multiple comparison test.

## Results

### Background performance

The growth performance results from the cattle during the background phase are shown in Table 2. The animals were fed the same rations during the background phase; the initial weight was no different between the steers and heifers but the ADG and final BW for the steers was higher than those for the heifers, but not significantly so (*P* > 0.05).

**Table 2:** Effects of sex on backgroundgrowth performance of cattle

**Item**	**Steer**	**Heifer**	**SEM**	***P***
Initial BW, kg	149.19	149.10	5.24	ns
Final BW, kg	299.61	288.05	7.99	ns
DMI, kg/d	5.04	5.10	0.02	ns
ADG, kg	0.82	0.74	0.03	ns
FCR	6.33	7.39	0.49	ns

Significance: ns not significant (*P* > 0.05).

### Finishing performance

The growth performance results from the cattle during the finishing phase are shown in Table 3. The initial BW values did not differ according to treatments or sex. The ADG and final BW were not affected by the energy or protein levels or by sex. The cattle fed an HE diet had a significantly lower dry matter intake (6.76 vs. 7.48 kgDM/d, *P* < 0.01) and FCR (Feed conversion ratio) (9.38 vs. 11.13, *P* < 0.01) than those fed an LE diet.

**Table 3:** Effects of dietary treatments and sex on finishing growth performance of cattle

**Item**		**HE**		**LE**		**SEM**	**E**	**P**	**S**
		**LP**	**HP**	**LP**	**HP**				
Initial BW, kg	M	319.75	286.50	299.60	294.00	16.86	ns	ns	ns
	F	293.50	283.66	295.20	282.83				
Final BW, kg	M	435.00	391.50	391.80	391.20	23.86	ns	ns	ns
	F	396.75	418.50	412.00	401.66				
DMI, kg/d	M	6.73^a^	6.74^a^	7.46^b^	7.47^b^	0.020	**	ns	ns
	F	6.74^a^	6.77^a^	7.50^b^	7.45^b^				
ADG, kg	M	0.77	0.70	0.62	0.65	0.079	ns	ns	ns
	F	0.69	0.90	0.78	0.79				
FCR	M	8.79	10.34	12.72	11.91	1.23	**	ns	ns
	F	10.13	8.23	9.72	10.15				

Significance: **(*P* < 0.01), ns not significant (*P* > 0.05).

M = male, steer, F = female, heifer, E = energy, P = protein, S = sex.

Interactions between energy, protein levels and sex were not significant so not shown in the table.

^ab^Means within the same row with the same superscript letter are not significantly different (*P* > 0.05).

The body measurements were not significantly different between treatments or sex at the start of the finishing phase (Table 4). Compared with cattle fed an LE diet, a greater chest girth increment (46.1 vs. 36.8 cm, *P* < 0.01) and larger chest girth (190.2 vs. 182.6 cm, *P* < 0.05) were found in cattle fed an HE diet with steers having a greater body length increment than heifers (9.0 vs. 8.3 cm, *P* < 0.01).

**Table 4:** Effects of dietary treatments and sex on body measurementsof cattle

**Item**		**HE**		**LE**		**SEM**	**E**	**P**	**S**
		**LP**	**HP**	**LE**	**HP**				
Start of finishing									
Chest girth, cm	M	144.9	145.7	147.1	147.0	2.00	ns	ns	ns
	F	142.3	143.4	146.5	142.8				
Withers height, cm	M	106,2	108.7	108.6	112.6	2.65	ns	ns	ns
	F	106.0	109.8	111.9	109.8				
Shin circumference, cm	M	18.4	16.6	17.8	16.1	0.88	ns	ns	ns
	F	15.3	17.8	16.3	15.4				
Body length, cm	M	123.9	116.3	122.1	122.2	3.81	ns	ns	ns
	F	119.3	121.1	122.5	118.5				
End of finishing									
Chest girth, cm	M	197.4	187.1	182.8	180.0	4.27	*	ns	ns
	F	184.0	192.2	183.7	183.9				
Withers height, cm	M	116.4	119.2	118.3	123.3	3.03	ns	ns	ns
	F	118.5	120.8	120.4	119.3				
Shin circumference, cm	M	19.9	18.5	19.7	18.2	0.92	ns	ns	ns
	F	17.2	19.3	17.8	17.1				
Body length, cm	M	132.8	125.0	130.8	131.6	3.71	ns	ns	ns
	F	127.2	129.5	130.7	127.0				
Increment									
Chest girth, cm	M	52.5	41.4	35.8	33.0	4.56	**	ns	ns
	F	41.7	48.7	37.2	41.1				
Withers height, cm	M	10.2	10.4	9.7	10.7	1.48	ns	ns	ns
	F	12.5	11.0	8.5	9.4				
Shin circumference, cm	M	1.5	1.9	1.9	2.1	0.21	ns	ns	ns
	F	1.8	1.6	1.6	1.8				
Body length, cm	M	8.9	8.7	8.8	9.4	0.34	ns	ns	**
	F	7.9	8.4	8.2	8.5				

Significance: *(*P* < 0.05), **(*P* < 0.01), ns not significant (*P* > 0.05).

M = male, steer, F = female, heifer, E = energy, P = protein, S = sex.

Effects of interactions between energy,protein levels and sexwere not significant (*P* > 0.05).

### Carcass characteristics

Thecarcass quality traits are shown in Table 5. The hot carcass weights were not affected by the energy or protein levels or by sex. The dressing percentage was higher with higher protein levels (*P* < 0.05) and the cattle in the LE treatment contained 3.4% units more lean meat and 3.6% units less fat than HE treatment (*P* < 0.05), which indicated that the meat:fat ratio was 23.6% higher. There was an energy × sex interaction for the 12^th^ rib fat thickness (*P* < 0.05). The bone content, meat:bone ratio and LM area were not affected by any of the factors. No differences were observed for the weight of most of the top and medium top grade cuts except for the highrib, striploin and chunk tender cuts which were affected by the energy or energy × protein interaction (Table 6). The yields of the top and medium top grade cuts were higher in the HE than LE treatments (*P* < 0.05).

**Table 5:** Effects of dietary treatments and sex oncarcass quality traits of cattle

**Item**		**HE**		**LE**		**SEM**	**E**	**P**	**S**	**E × P**	**E × S**
		**LP**	**HP**	**LP**	**HP**						
HCW, kg	M	235.50	214.75	205.00	210.00	12.284	ns	ns	ns	ns	ns
	F	207.25	233.75	223.60	220.83						
Dressing percentage, %	M	54.00	54.75	52.00	53.80	0.009	ns	*	ns	ns	ns
	F	52.75	55.83	54.60	55.00						
CCW, kg	M	231.00	210.50	201.00	205.80	11.80	ns	ns	ns	ns	ns
	F	203.00	229.16	219.20	216.33						
Carcass composition, %											
Meat	M	64.29	67.65	71.08	70.47	0.018	*	ns	ns	ns	ns
	F	62.78	67.21	69.05	67.48						
Fat	M	19.51	19.56	14.05	13.99	0.017	*	ns	*	ns	ns
	F	22.23	18.11	17.34	19.84						
Bone	M	15.29	11.89	14.15	14.68	0.143	ns	ns	ns	ns	ns
	F	13.53	14.38	12.73	12.22						
Meat:fat ratio	M	3.30	3.45	5.04	5.04	0.466	*	ns	*	ns	*
	F	2.83	3.71	3.99	3.41						
Meat:bone ratio	M	4.20	5.69	5.01	4.80	0.421	ns	ns	ns	ns	ns
	F	4.65	4.67	5.44	5.53						
Fat thickness, cm	M	1.10	0.98	0.72	0.71	0.118	ns	ns	ns	ns	*
	F	0.76	1.04	1.01	0.86						
LM area, cm^2^	M	65.71	66.59	54.24	60.47	3.660	ns	ns	ns	ns	ns
	F	59.63	59.04	59.59	58.31						

Significance: *(*P* < 0.05), ns not significant (*P* > 0.05).

M = male, steer, F = female, heifer, E = energy, P = protein, S = sex.

Effects of P × S and E × P × Sinteractions were not significant (*P* > 0.05).

**Table 6:** Effects of diets and sex on yield of Top and Medium top grade commercial cuts

**Item**	**HE**		**LE**		**SEM**	**E**	**P**	**E × P**
	**LP**	**HP**	**LP**	**HP**				
Total meat, kg	138.3	148.9	146.9	145.6	11.6	ns	ns	ns
Top grade cuts								
Highrib, kg	8.1	8.3	6.9	7.2	0.38	*	ns	ns
Ribeye, kg	8.3	8.3	7.8	7.5	0.50	ns	ns	ns
Striploin, kg	5.4	6.4	6.2	5.7	0.30	ns	ns	*
Tenderloin, kg	2.7	2.4	2.4	2.3	0.14	ns	ns	ns
Medium top grade cuts								
Chunk tender, kg	2.1	1.8	1.7	2.0	0.10	ns	ns	*
Topside, kg	9.6	9.3	8.2	8.4	0.59	ns	ns	ns
Outside flat, kg	5.0	4.8	4.6	4.7	0.28	ns	ns	ns
Eye round, kg	3.0	2.9	2.8	2.8	0.18	ns	ns	ns
Rump, kg	5.5	6.1	5.4	4.7	0.36	ns	ns	ns
Knuckle, kg	6.9	6.3	6.9	7.0	0.37	ns	ns	ns
Top grade cuts yield, %	18.0	17.3	15.9	15.6	0.006	*	ns	ns
Medium top grade cuts yield, %	23.5	21.0	20.1	20.3	0.009	*	ns	ns
Total, %	41.4	38.3	36.3	36.7	0.01	*	ns	ns

Significance: *(*P* < 0.05), ns not significant (*P* > 0.05).

M = male, steer, F = female, heifer, E = energy, P = protein.

Sex had no effect on yield of Top and Medium top grade commercial (*P* > 0.05).

The carcass measurements are shown in Table 7. The steers had a greater chest depth and maximum leg girth (*P* < 0.05) and a thinner rib lean thickness (*P* < 0.01) than the heifers. The cattle fed an HE diet had a greater leg and rib lean thickness than those fed an LE diet (*P* < 0.01).

**Table 7:** Effects of dietary treatments and sex on carcass measurements of cattle

**Item**		**HE**		**LE**		**SEM**	**E**	**P**	**S**
		**LP**	**HP**	**LP**	**HP**				
Carcass length, cm	M	137.0	127.5	131.8	135.0	3.18	ns	ns	ns
	F	128.0	136.3	133.7	132.5				
Chest depth, cm	M	70.3	67.9	66.9	68.0	1.31	ns	ns	*
	F	64.2	68.0	64.9	66.8				
Maximum leg, cm	M	73.4	72.2	73.0	73.6	1.64	ns	ns	*
	F	66.8	71.0	71.6	70.9				
Leg length, cm	M	62.9	62.8	63.9	64.3	1.57	ns	ns	ns
	F	61.8	63.3	63.6	62.5				
Leg lean thickness, cm	M	10.2	11.2	10.4	9.7	0.40	**	ns	ns
	F	10.4	10.9	9.3	9.4				
Loin lean thickness, cm	M	6.0	6.1	6.8	6.0	0.34	ns	ns	ns
	F	6.0	7.0	5.9	6.4				
Rib lean thickness, cm	M	4.4^ab^	5.0^ab^	4.1^b^	4.0^ab^	0.29	**	ns	**
	F	5.0^ab^	5.8^a^	5.0^ab^	4.8^b^				

Significance: *(*P* < 0.05), **(*P* < 0.01), ns not significant (*P* > 0.05).

M = male, steer, F = female, heifer, E = energy, P = protein, S = sex.

Effects of interactions between energy, protein level and sex were not significant (*P* > 0.05).

^ab^Means within same row with the same superscript letter are not significantly different (*P* > 0.05).

### Meat quality

The LM chemical composition and quality traits are shown in Tables 8 and 9. The dry matter was higher for heifers than for steers (29.2 vs. 27.6%, *P* < 0.05). The ultimate pH, protein and intramuscular fat content were significantly affected by the energy level, with a lower ultimate pH (5.71 vs. 5.79, *P* < 0.01), protein content (68.6 vs. 74.9% DM, *P* < 0.05) and higher intramuscular fat content (29.9 vs. 22.8% DM, *P* < 0.01) detected in the HE treatment compared with the LE treatment. The shear force value, drip loss, cooking loss and water holding capacity were not affected by the energy or protein levels or by sex and averaged 3.14 kg, 2.5, 31.5 and 52.9%, respectively. The fatty acid composition (Table 10) and amino acid composition (Tables 11 and 12) were not influenced by the energy or protein levels or by sex. (*P* > 0.05), but the ratio of unsaturated fatty acids to saturated fatty acids was higher in the HE treatment compared with the LE treatment (*P* < 0.05). The percentage of amino acids producing an umami, sour or sweet taste was more than 60% with the percentage producing a bitter taste at about 29%. The essential and non-essential amino acids were approximately 28.4 and 71.7%, respectively.

**Table 8:** Effects of dietary treatments and sex onchemical composition of LM

**Item**		**HE**		**LE**		**SEM**	**E**	**P**	**S**
		**LP**	**HP**	**LP**	**HP**				
Dry matter, %	M	28.6	28.0	27.7	26.2	0.010	ns	ns	*
	F	30.2	29.3	29.0	28.3				
Crude protein, % DM	M	70.2	70.8	76.8	75.8	0.035	*	ns	ns
	F	65.2	68.0	72.7	74.4				
Intramuscular fat, % DM	M	28.4	27.7	18.5	22.4	0.038	**	ns	ns
	F	32.3	31.2	25.7	24.6				

Significance: *(*P* < 0.05), **(*P* < 0.01), ns not significant (*P* > 0.05).

M = male, steer, F = female, heifer, E = energy, P = protein, S = sex.

Effects of interactions between energy, protein level and sex were not significant (*P* > 0.05).

**Table 9:** Effects of dietary treatments and sex onquality traits of LM

**Item**		**HE**		**LE**		**SEM**	**E**	**P**	**S**
		**LP**	**HP**	**LP**	**HP**				
pH	M	5.67^b^	5.77^ab^	5.74^ab^	5.80^ab^	0.042	**	ns	ns
	F	5.75^ab^	5.65^b^	5.76^ab^	5.86^a^				
Shear force, kg	M	3.38	3.31	2.81	3.11	0.370	ns	ns	ns
	F	3.34	2.98	3.40	2.82				
Cooking loss, %	M	30.40	31.33	32.50	33.20	0.016	ns	ns	ns
	F	30.33	32.50	30.33	31.14				
Drip loss, %	M	2.60	2.00	2.50	2.13	0.013	ns	ns	ns
	F	2.00	2.66	2.66	3.24				
WHC, %	M	53.00	51.16	54.00	53.80	0.019	ns	ns	ns
	F	50.66	52.50	53.83	54.43				

Significance: **(*P* < 0.01), ns not significant (*P* > 0.05).

M = male, steer, F = female, heifer, E = energy, P = protein, S = sex.

Effects of interactions between energy, protein level and sex were not significant (*P* > 0.05).

^ab^Means within same row with the same superscript letter are not significantly different (*P* > 0.05).

**Table 10:** Effects of dietary treatments and sex onfatty acid composition of LM (mg/g DM)

**Item**	**HE**		**LE**		**SEM**	**E**	**P**	**E × P**
	**LP**	**HP**	**LP**	**HP**				
C14:0	6.70	5.45	4.56	5.88	1.31	ns	ns	ns
C14:1	1.97	2.12	1.71	2.26	0.36	ns	ns	ns
C16:0	61.68	51.55	47.46	55.03	9.28	ns	ns	ns
C16:1	13.86	10.40	9.18	10.72	1.96	ns	ns	ns
C18:0	22.92	19.72	17.06	21.42	3.66	ns	ns	ns
C18:1^trans-9^	2.14	1.26	1.35	1.24	0.37	ns	ns	ns
C18:1 ^cis-9^	91.91	75.95	63.24	76.70	13.79	ns	ns	ns
C18:2 ^cis-9,12^	4.27	4.61	3.32	3.22	0.80	ns	ns	ns
C18:3n-3	0.30	0.53	0.23	0.46	0.14	ns	ns	ns
C20:3n-6	2.75	2.22	2.00	2.95	0.24	ns	ns	**
SFA	91.30	76.72	69.08	82.33	14.11	ns	ns	ns
MUFA	109.9	89.72	75.48	90.93	16.19	ns	ns	ns
PUFA	7.16	7.08	5.39	6.38	0.85	ns	ns	ns
n-6:n-3	25.94	19.74	14.95	17.36	7.99	ns	ns	ns
P:S	0.11	0.14	0.09	0.11	0.03	ns	ns	ns
UFA:SFA	1.31	1.27	1.17	1.20	0.05	*	ns	ns

Significance: *(*P* < 0.05), **(*P* < 0.01), ns not significant (*P* > 0.05).

M = male, steer, F = female, heifer, E = energy, P = protein, S = sex.

Sex had no effect on fatty acid composition of LM (*P* > 0.05).

**Table 11:** Effects of dietary treatments and sex onamino acid composition of LM (mg/100 mg DM basis)

**Item**	**HE**		**LE**		**SEM**	**E**	**P**	**E × P**
	**LP**	**HP**	**LP**	**HP**				
Essential								
Lysine	4.61	3.72	4.06	4.25	0.46	ns	ns	ns
Valine	3.53	3.05	2.94	2.94	0.35	ns	ns	ns
Histidine	1.26	0.64	0.74	0.64	0.27	ns	ns	ns
Leucine	4.82	4.65	4.61	4.76	0.19	ns	ns	ns
Isoleucine	3.19	3.37	3.05	3.14	0.18	ns	ns	ns
Methionine	4.28	4.11	3.90	4.03	0.17	ns	ns	ns
Phenylalanine	1.59	1.58	1.61	1.56	0.12	ns	ns	ns
Threonine	4.52	4.09	4.16	4.18	0.27	ns	ns	ns
Total E	27.80	25.21	25.09	25.50	0.97	ns	ns	ns
Non essential								
Aspartic acid	13.86	13.76	13.35	12.15	1.44	ns	ns	ns
Glutamic acid	21.93	19.54	18.67	20.41	1.19	ns	ns	*
Cysteine	4.79	4.59	4.50	4.45	0.33	ns	ns	ns
Alanine	5.99	5.53	5.36	5.70	0.32	ns	ns	ns
Glycine	4.92	4.32	4.29	4.38	0.32	ns	ns	ns
Serine	9.21	6.23	5.86	5.76	1.37	ns	ns	ns
Proline	2.14	2.13	2.02	2.08	0.21	ns	ns	ns
Arginine	6.26	5.20	5.23	5.28	0.48	ns	ns	ns
Tyrosine	3.82	2.94	3.41	3.54	0.42	ns	ns	ns
Total NE	72.93	64.25	62.69	63.75	2.76	ns	ns	ns
Total AA	100.73	89.46	87.79	89.25	3.51	ns	ns	ns
E/NE, %	38.12	40.00	40.02	40.58	0.015	ns	ns	ns
E/TAA, %	27.59	28.38	28.57	28.77	0.007	ns	ns	ns

Significance: *(*P* < 0.05), ns not significant (*P* > 0.05).

M = male, steer, F = female, heifer, E = energy, P = protein.

Sex had no effect on amino acid composition of LM (*P* > 0.05).

**Table 12:** Effects of dietary treatments and sex onflavor amino acid composition of LM (mg/100 mg DM basis)

**Item**	**HE**		**LE**		**SEM**	**E**	**P**	**E × P**
	**LP**	**HP**	**LP**	**HP**				
Lysine	4.61	3.72	4.06	4.25	0.46	ns	ns	ns
Cysteine	4.79	4.59	4.50	4.45	0.33	ns	ns	ns
Umami taste								
Aspartic acid	13.86	13.76	13.35	12.15	1.44	ns	ns	ns
Glutamic acid	21.93	19.54	18.67	20.41	1.19	ns	ns	*
Total AA(U)	35.79	33.30	32.02	32.56	1.71	ns	ns	ns
Sweet taste								
Threonine	4.52	4.09	4.16	4.18	0.27	ns	ns	ns
Alanine	5.99	5.53	5.36	5.70	0.32	ns	ns	ns
Glycine	4.92	4.32	4.29	4.38	0.32	ns	ns	ns
Serine	9.21	6.23	5.86	5.76	1.37	ns	ns	ns
Proline	2.14	2.13	2.02	2.08	0.21	ns	ns	ns
Total AA(S)	26.79	22.30	21.69	22.10	1.41	ns	ns	ns
Bitter taste								
Arginine	6.26	5.20	5.23	5.28	0.48	ns	ns	ns
Histidine	1.26	0.64	0.74	0.64	0.27	ns	ns	ns
Leucine	4.82	4.65	4.61	4.76	0.19	ns	ns	ns
Isoleucine	3.19	3.37	3.05	3.14	0.18	ns	ns	ns
Methionine	4.28	4.11	3.90	4.03	0.17	ns	ns	ns
Phenylalanine	1.59	1.58	1.61	1.56	0.12	ns	ns	ns
Tyrosine	3.82	2.94	3.41	3.54	0.42	ns	ns	ns
Valine	3.53	3.05	2.94	2.94	0.35	ns	ns	ns
Total AA(B)	28.74	25.54	25.52	25.89	1.21	ns	ns	ns
Total AA	100.73	89.46	87.79	89.25	3.51	ns	ns	ns
AA(U)/TAA, %	36.33	36.82	36.48	36.14	0.01	ns	ns	ns
AA(S) /TAA, %	26.09	25.12	24.71	24.86	0.007	ns	ns	ns
AA(B) /TAA, %	28.33	28.79	29.06	29.30	0.009	ns	ns	ns

Significance: *(*P* < 0.05), ns not significant (*P* > 0.05).

M = male, steer, F = female, heifer, E = energy, P = protein.

Sex had no effect on amino acid composition of LM (*P* > 0.05).

## Discussion

### Growth performance

The initial BW (293.4 kg) did not differ between treatments or sex at the beginning of the finishing phase, and the ADG was not affected by the energy or protein levels or sex averaging 0.74 kg/d. The cattle fed an HE diet had a lower dry matter intake (6.76 vs. 7.48 kgDM/d, *P* < 0.01) and FCR (9.38 vs. 11.13, *P* < 0.01) compared with those fed an LE diet. The DMI was lower in the HE treatment group, which can be explained by the theory of satiety limit intake where in the metabolic needs are completely met [17]. Compared with the current study, Angus crossbred cattle such as Angus × Gelbvieh gained 1.76 kg/d and their FCR was 5.36 when they were fed for 180 d with a similar dietary nutrition level at nearly the same initial BW of approximately 293.6 kg [8]. The contrast could be considered the result of the genetic influence of the Xiangxi yellow cattle because the rate of gain is usually positively related to the mature size [18]. The mature weight and withers height of the Xiangxi yellow bulls were 334.3 kg and 117.1 cm, respectively, and for cows were 240.2 kg and 106.1 cm, respectively [2]. However, the mature weight and withers height of the Gelbvieh bulls were 1,100–1,300 kg and 148–156 cm, respectively, and of the cows, 650–850 kg and 140 cm, respectively [19]. Therefore, the extremely large difference between the mature size ofthe Xiangxi yellow cattle and Gelbvieh cattle resulted in a lower ADG for the Angus × Chinese Xiangxi yellow cattle compared with the Angus × Gelbvieh. However, growth stimulants were not used for the cattle in the present study, whereas Synovex-S was implanted in the Angus × Gelbvieh cross, which might have improved the ADG in the research of Ludden et al. [8].

The average withers height at 12 and 18mon for the cattle in the present study was 109.2 and 119.5 cm, respectively, which was shorter than the Angus × Hereford steers that had a yearling height of 112.0 and 122.4 cm at the age of 16 mon [20]. Angus bulls can reach a height of 120.2 cm at 12 mon [21]. Withers height, shin circumference and body length are mainly determined by the composition of the bones, which are an early maturing part of the body; however the chest girth is a relatively late maturing part of the body and is mainly determined by meat and fat. Therefore the chest increment revealed that the higher energy in the diet may have resulted in additional protein deposition and fat cover.

### Carcass characteristics

Carcass quality traits are shown in Table 5. The hot carcass weights were not affected byenergy or protein levels or by sex and had a mean value of 219.0 kg. This result cannot be compared with data obtained from Angus or other Angus crossbred cattle, because they have a greater growth rate resulting in a heavier slaughter and carcass weight at the age of 17–19 mon [6,21] or even at 14 mon [22]. The authors reported carcass weights of 292.3 and 335.7 kg for the Angus bulls and 293.8 kg for 19 various Angus crossbred steers. The dressing percentage was higher with increasing protein levels (53.4 vs. 54.9%, *P* < 0.05), which might have been caused by theincreased water concentrations in tissues as a result of the hydrophilic characteristics of systemic ammonium ions leading to higher dressing percentage [23]. The mean dressing percentage for all of the cattle was 54.2%, which was lower than the values of 55.0, 56.2 and 58.2% found by Cuvelier et al. [6], Albertí et al. [21] and Laborde et al. [22], respectively. In China, a dressing percentage of 52% is set at a threshold value for the gain or loss of 0.3 Yuan RMB per kg for one percent higher or lower [24].

Regarding the carcass composition, the cattle in the LE diet treatment contained more lean meat (69.6 vs. 65.5%, *P* < 0.05) and a lower fat content (16.3 vs. 19.9%, *P* < 0.05) than in the HE treatment, which might have resulted from the higher glucose content in the HE diet which increased the fat deposition. In the present study, a higher meat content (67.6 vs. 62.2%, 61.6%) and lower fat content (18.1 vs. 23.6%, 21.7%) was observed compared with that found by Cuvelier et al. [6] and Albertí et al. [21] at a slaughter age of 17-19 mon because the age of puberty for Angus cattle is 295 d [25], which is less than that of Chinese Xiangxi yellow cattle at 497 d [2]. Therefore, pure Angus cattle deposit fat at a younger age and the high growth rates of 1.66 kg/d and 1.9 kg/d, reported by Cuvelier et al. [6] and Albertí et al. [21], accelerates fat deposition. Thus, the fat contents in these previous experiments were higher than that of the Angus × Chinese Xiangxi yellow cattle in the present study.

Heifers had a higher fat content than steers (16.8 vs. 19.4%, respectively) and under the conditions of an LE diet, heifers had a greater 12^th^ rib fat thickness than steers. This suggests that heifers deposit fat more easily, which is possibly related to hormonal effects [26]. The LM area was not different between treatments or sex and averaged 60.4 cm^2^ which was within the range of 58.7–70.3 cm^2^ for pure Angus or Angus crossbred cattle with a slaughter weight of approximately 400 kg [27-29].

The yields of top and medium top grade cuts were higher in the HE treatment than in the LE treatment, which suggests that the high plane of nutrition, especially for the dietary energy level, contributed to the higher yields of the top and medium top grade cuts.

Steers had a greater chest depth and maximum leg girth, but thinner rib lean thickness (*P* < 0.01) than heifers. Although the male cattle had been castrated before the experiment, they still had more development in the fore body and legs than the heifers. The increasing energy level contributed to a greater leg and rib lean thickness.

### Meat quality

The DM of the LM was higher for heifers than steers, which can be explained by the heifers having a greater intramuscular fat content, because fat tissues contain little water, so the DM of the LM was higher [30]. A higher intramuscular fat content (29.9 vs. 22.8%, *P* < 0.01) and lower protein content (68.6 vs. 74.9%, *P* < 0.05) were observed in the HE treatment compared with the LE diet treatment, this could have resulted from the intramuscular fat being derived from a glucose substrate that is absorbed in the small intestine and stimulates a greater activity of ATP citrate lyase, which synthesizes fat from glucose [31]. A maize-based diet could enhance the glucose absorbed in the small intestine. In the present study, a greater amount of ground corn was included in the ration in the HE compared with the LE treatment (63.8 vs.39.0%), which resulted ina higher intramuscular fat content from the HE diet treatment. The intramuscular fat content was 26.4% in the present study, which was higher than the value of 21% for Angus steers [32] and 9.3% for Angus × Limousin steers found in previous studies [7]. However, the fat thickness was less than that found for Angus and Angus × Limousin (0.90 vs. 1.15 cm, 0.98 cm). This result suggests that F1 Angus × Chinese Xiangxi yellow cattle develop intramuscular fat more strongly at lower levels of subcutaneous fat.

Post-slaughter, glycogen is converted to lactic acid and there is an associated reduction in muscle pH from the neutral value of 7.2 [33]. The ultimate pH in this experiment was lower (5.71 vs. 5.79, *P* < 0.01) for the cattle fed an HE diet compared with an LE diet, because there is an increasing effect of energy level with increased glycogen availability. The ultimate pH can also affect meat tenderness, with a pH of 5.4–5.8 found in normal, tender meat, a pH value of 5.8–6.2 in inconsistently tender meat (moderate DFD) and pH > 6.2 found intender meat with microbial spoilage (DFD meat) [34]. Therefore, the meat observed in the present study can be considered as normal, tender meat with values of meat pH values similar to those reported by Cuvelier et al. [6] and Faucitano et al. [7].

Meat tenderness is the most important quality trait for the consumer and consumers prefer and will pay more for tender beef meat [35]. A threshold shear force of 4.6 kg has been used to distinguish tough and tender steaks [36]. A shear force value from 2.27 to 3.58 kg is considered tender; 4.08–5.40 kg intermediate; and 5.90–7.21 kg tough [37]. The shear force value was not affected by the energy or protein levels or sex with a mean value of 3.14 kg, so can be classified as “very tender” meat. One study has found that the shear force value of meat was 3.62 kg when Angus steers were slaughtered at a younger age of 14 mon with a 14 d post-mortem [38], this value was higher than that found in the present study. A younger slaughter age and longer post-mortem ageing time could produce more tender meat [39,40]. Therefore, if F1 Angus × Chinese Xiangxi yellow cattle were slaughtered at the age of 14 mon with 14 d post-mortem ageing, the meat would be more tender.

Drip loss can be categorized as follows: low drip loss ≤2.60%, medium drip loss: 2.60–4.00%, and high drip loss ≥4.00% [41]. The average drip loss in the present study was 2.5%, which is thus classified as low. The cooking loss was 31.5%, a value similar to 33.8% for Angus bulls [6] and 29.5% for Angus × Limousin cattle [7]. The WHC increased slightly in the LE diet treatment, which could be related to the “sponge effect” hypothesis, in which the higher ultimate pH of the LE treatment accelerates the breakdown of meat structure and results in a reduction of water loss from the channels [42].

### Nutritive profile (intramuscular fatty acid and amino acid composition)

The intramuscular fatty acid content (FA) was not significantly different between treatments or sex and was dominated by MUFA at 51.4%, followed by SFA at approximately 44.9% and PUFA at approximately 3.7%. The mean value of the n-6:n-3 ratio was 19.5 which was much higher than the <4.0 value from nutritional advice [43]. When the cattle were grain-fed, the concentrate diet could have improved the proportion of PUFA, which was dominated by n-6, especially C18:2n-6. Forages such as fresh grass or grass silage are rich in C18:3n-3 [44]. Therefore, grass-fed cattle have a higher amount of C18:3n-3 and a lower amount of C18:2n-6 in their muscles compared with concentrate-fed cattle [45]. The cattle in the present study were fed a high concentrate diet and the roughage was yellow rice straw instead of fresh grass or silage, thus a higher ratio of n-6:n-3 was observed. The mean value of the P:S ratio was 0.11, which is normal for beef [46]. These results were consistent with the results of Warren et al. [9] and Ludden et al. [8]. The UFA/SFA ratio was significantly higher in the HE treatment, which was verified in this study and is related to a loss of efficiency of rumen biohydrogenation because less fibrous diets pass through the rumen at a faster rate. From the perspective of meat flavor, ‘sweet’, ‘oily’, ‘chemical-like’ and ‘perfume-like’ are induced by a high C18:2n-6 content in the meat [47] and ‘fishy’ and ‘grassy’ flavors by higher n-3 content [48]. Therefore, the meat in the present study would taste ‘sweet’, ‘oily’, ‘chemical-like’ and ‘perfume-like’ and not include ‘fishy’ and ‘grassy’ flavors.

The amino acid composition in this study was not affected by the energy or protein levels or by sex. The percentage of amino acids producing the tastes of umami, sour and sweet was more than 60% with the percentage producing a bitter taste at approximately 29%. The essential and non-essential amino acid requirements of an adult man are 0.18 g/kg per day (EAA) and 0.48 g/kg per day (NEAA), respectively, which equals EAA/NEAA = 37.5% and EAA/TAA = 27.3% [49]. In the present study, the mean ratios of EAA/NEAA and EAA/TAA of the meat samples were 39.7 and 28.4%, which were a little higher than those recommended by FAO/WHO/UNU [48] but can meet an adult man’s needs appropriately, therefore the meat appears to be an excellent source of high biological value protein.

## Conclusions

The cattle carcass characteristics and chemical composition of the meat were significantly influenced by dietary energy and protein levels and by sex. The growth performance, meat quality traits and nutritive profiles were not affected by energy or protein levels or by sex.

The meat quality of the F1 Angus × Chinese Xiangxi yellow cattle was high based on its tenderness, flavor and nutritional value. However, there is considerable potential to obtain higher daily gains and meat production for this kind of crossbred cattle. However, feeding and breeding techniques must be developed to determine the best methods for improving beef products in both quantitative and qualitative terms.

## Competing interests

The authors declare that they have no competing interests.

## Authors’ contributions

LYL carried out the experiments, finished the data analysis and drafted the manuscript. YKZ, XYW and YH participated in the experiments and helped with data collection and analysis. BHC conceived the experiment and finished the manuscript. All authors approved the final version of the manuscript for publication.

## References

[B1] MOA National Protection List of Livestock and Poultry Genetic Resources Ministry of Agriculture of the People's Republic of China 2006 662 245

[B2] Ouyang SJ Livestock and poultry breeds of Hunan Province 1984 Changsha: Hunan Science and Technology Press 54 60

[B3] Dunshea FR D’Souza DN Pethick DW Harper GS Warner RD Effects of dietary factors and other metabolic modifiers on quality and nutritional value of meat Meat Science 2005 71 8 38 10.1016/j.meatsci.2005.05.001 22064049

[B4] Arthaud VH Mandigo RW Koch RM Kotula AW Carcass composition, quality and palatability attributes of bulls and steers fed different energy levels and killed at four ages J Anim Sci 1970 44 53 64 10.2527/jas1986.63192x 3733582

[B5] Kannan G Gadiyaram KM Galipalli S Carmichael A Kouakou B Pringle TD McMillin KW Gelaye S Meat quality in goats as influenced by dietary protein and energy levels, and postmortem aging Small Ruminant Research 2006 61 45 52 10.1016/j.smallrumres.2005.01.006

[B6] Cuvelier C Cabaraux JF Dufrasne I Clinquart A Hocquette JF Istasse L Hornick JL Performance, slaughter characteristics and meat quality of young bulls from Belgian Blue, Limousin and Aberdeen Angus breeds fattened with a sugar-beet pulp or a cereal-based diet Animal Science 2007 82 125 132

[B7] Faucitano L Berthiaume R D'Amours M Pellerin D Ouellet DR Effects of corn grain particle size and treated soybean meal on carcass and meat quality characteristics of beef steers finished on a corn silage diet Meat Sci 2011 88 750 754 10.1016/j.meatsci.2011.03.008 21454022

[B8] Ludden PA Kucuk O Rule DC Hess BW Growth and carcass fatty acid composition of beef steers fed soybean oil for increasing duration before slaughter Meat Sci 2009 82 185 192 10.1016/j.meatsci.2009.01.009 20416764

[B9] Warren HE Scollan ND Enser M Hughes SI Richardson RI Wood JD Effects of breed and a concentrate or grass silage diet on beef quality in cattle of 3 ages. I: Animal performance, carcass quality and muscle fatty acid composition Meat Sci 2008 78 256 269 10.1016/j.meatsci.2007.06.008 22062278

[B10] AOAC Official methods of analysis Association of Official Analytical Chemists 2000 17 Arlington, VA

[B11] Van Soest PJ Robertson JB Lewis BA **Methods for Dietary Fiber, Neutral Detergent Fiber, and Nonstarch Polysaccharides in Relation to Animal Nutrition** J Dairy Sci 1991 74 3583 3597 10.3168/jds.S0022-0302(91)78551-2 1660498

[B12] Boer HD Dumont B Pomeroy R Weniger J Manual on E.A.A.P. Reference Methods for the assessment of carcass characteristics in cattle Livestock Production Science 1974 1 151 164 10.1016/0301-6226(74)90055-4

[B13] Verbeke R Van de Voorde G Détermination de la composition de demi-carcasses de bovins par la dissection d'une seule côte Revue de l'Agriculture 1978 31 875 880

[B14] Chen YC Discussions on the names of beef carcass high-grade cuts J Yellow Cattle Sci 2003 29 1 3

[B15] Lepage G Roy C Direct transesterification of all classes of lipids in a one-step reaction J Lipid Res 1986 27 114 120 3958609

[B16] Wu G Davis PK Flynn NE Knabe DA Davidson JT Endogenous synthesis of arginine plays an important role in maintaining arginine homeostasis in postweaning growing pigs J Nutr 1997 127 2342 2349 9405584 10.1093/jn/127.12.2342

[B17] Grovum WL Mechanisms explaining the effects of short chain fatty acids on feed intake in ruminants-osmotic pressure, insulin and glucagon Ruminant physiology: digestion, metabolism, growth and reproduction. Proceedings 8th International Symposium on Ruminant Physiology 1995 173 197

[B18] Klosterman EW Beef cattle size for maximum efficiency J Anim Sci 1972 34 875 880

[B19] Felius M Cattle breeds - an encyclopedia Misset.,Doetinchem 1995 Netherlands: 1995

[B20] Paschal J Sanders J Kerr J Lunt D Herring A Postweaning and feedlot growth and carcass characteristics of Angus-, gray Brahman-, Gir-, Indu-Brazil-, Nellore-, and red Brahman-sired F1 calves J Anim Sci 1995 73 373 380 7601767 10.2527/1995.732373x

[B21] Albertí P Panea B Sañudo C Olleta JL Ripoll G Ertbjerg P Christensen M Gigli S Failla S Concetti S Hocquette JF Jailler R Rudel S Renand G Nute GR Richardson RI Williams JL Live weight, body size and carcass characteristics of young bulls of fifteen European breeds Livestock Science 2008 114 19 30 10.1016/j.livsci.2007.04.010

[B22] Laborde FL Mandell IB Tosh JJ Wilton JW Buchanan-Smith JG Breed effects on growth performance, carcass characteristics, fatty acid composition, and palatability attributes in finishing steers J Anim Sci 2001 79 355 365 11219444 10.2527/2001.792355x

[B23] Gleghorn JF Elam NA Galyean ML Duff GC Cole NA Rivera JD Effects of crude protein concentration and degradability on performance, carcass characteristics, and serum urea nitrogen concentrations in finishing beef steers J Anim Sci 2004 82 2705 2717 15446487 10.2527/2004.8292705x

[B24] Zhou GH Liu L Xiu XL Jian HM Wang LZ Sun BZ Tong BS Productivity and carcass characteristics of pure and crossbred Chinese Yellow Cattle Meat Sci 2001 58 359 362 10.1016/S0309-1740(00)00160-1 22062425

[B25] Lunstra DD Echternkamp SE Puberty in beef bulls: acrosome morphology and semen quality in bulls of different breeds J Anim Sci 1982 55 638 7130067 10.2527/jas1982.553638x

[B26] Morgan JB Wheeler TL Koohmaraie M Crouse JD Savell JW Effect of castration on myofibrillar protein turnover, endogenous proteinase activities, and muscle growth in bovine skeletal muscle J Anim Sci 1993 71 408 414 7680026 10.2527/1993.712408x

[B27] Arthur P Archer J Johnston D Herd R Richardson E Parnell P Genetic and phenotypic variance and covariance components for feed intake, feed efficiency, and other postweaning traits in Angus cattle J Anim Sci 2001 79 2805 2811 11768108 10.2527/2001.79112805x

[B28] Urick JJ MacNEIL MD Reynolds WL Biological type effects on postweaning growth, feed efficiency and carcass characteristics of steers J Anim Sci 1991 69 490 497 2016178 10.2527/1991.692490x

[B29] Henricks DM Jenkins TC Ward JR Krishnan CS Grimes L Endocrine responses and body composition changes during feed restriction and realimentation in young bulls J Anim Sci 1994 72 2289 2297 8002448 10.2527/1994.7292289x

[B30] Dinh TTN Blanton JR Riley DG Chase CC Coleman SW Phillips WA Brooks JC Miller MF Thompson LD Intramuscular fat and fatty acid composition of longissimus muscle from divergent pure breeds of cattle J Anim Sci 2009 88 756 766 19783694 10.2527/jas.2009-1951

[B31] Pethick DW McIntyre BL Tudor G Rowe JB The partitioning of fat in ruminants: can nutrition be used as a tool to regulate marbling? Recent Advances in Animal Nutrition in Australia 1997 11 151 158

[B32] Baker S Szasz J Klein T Kuber P Hunt C Glaze J Falk D Richard R Miller J Battaglia R Residual feed intake of purebred Angus steers: Effects on meat quality and palatability J Anim Sci 2006 84 938 945 16543572 10.2527/2006.844938x

[B33] Muir PD Deaker JM Bown MD **Effects of forage**‒ **and grain**‒**based feeding systems on beef quality: A review** New Zealand Journal of Agricultural Research 1998 41 623 635 10.1080/00288233.1998.9513346

[B34] Silva J Patarata L Martins C Influence of ultimate pH on bovine meat tenderness during ageing Meat Sci 1999 52 453 459 10.1016/S0309-1740(99)00029-7 22062710

[B35] Killinger KM Calkins CR Umberger WJ Feuz DM Eskridge KM Consumer sensory acceptance and value for beef steaks of similar tenderness, but differing in marbling level J Anim Sci 2004 82 3294 3301 15542476 10.2527/2004.82113294x

[B36] Shackelford SD Morgan JB Cross HR Savell JW Iidentification of threshold levels for warner‒bratzler shear force in beef top loin steaks J Muscle Foods 1991 2 289 296 10.1111/j.1745-4573.1991.tb00461.x

[B37] Boleman S Boleman S Miller R Taylor J Cross H Wheeler T Koohmaraie M Shackelford S Miller M West R Consumer evaluation of beef of known categories of tenderness J Anim Sci 1997 75 1521 1524 9250512 10.2527/1997.7561521x

[B38] Loken BA Maddock RJ Stamm MM Schauer CS Rush I Quinn S Lardy GP Growing rate of gain on subsequent feedlot performance, meat, and carcass quality of beef steers J Anim Sci 2009 87 3791 3797 10.2527/jas.2009-1853 19717783

[B39] Purchas RW Burnham DL Morris ST Effects of growth potential and growth path on tenderness of beef longissimus muscle from bulls and steers J Anim Sci 2002 80 3211 3221 12542162 10.2527/2002.80123211x

[B40] Marino R Albenzio M Malva A d Santillo A Loizzo P Sevi A Proteolytic pattern of myofibrillar protein and meat tenderness as affected by breed and aging time Meat Sci 2013 95 281 287 10.1016/j.meatsci.2013.04.009 23743033

[B41] Traore S Aubry L Gatellier P Przybylski W Jaworska D Kajak-Siemaszko K Santé-Lhoutellier V Higher drip loss is associated with protein oxidation Meat Sci 2012 90 917 924 10.1016/j.meatsci.2011.11.033 22193037

[B42] Farouk MM Mustafa NM Wu G Krsinic G The “sponge effect” hypothesis: An alternative explanation of the improvement in the waterholding capacity of meat with ageing Meat Sci 2012 90 670 677 10.1016/j.meatsci.2011.10.012 22104253

[B43] Scollan N Hocquette J-F Nuernberg K Dannenberger D Richardson I Moloney A Innovations in beef production systems that enhance the nutritional and health value of beef lipids and their relationship with meat quality Meat Sci 2006 74 17 33 10.1016/j.meatsci.2006.05.002 22062713

[B44] Dewhurst RJ Shingfield KJ Lee MRF Scollan ND Increasing the concentrations of beneficial polyunsaturated fatty acids in milk produced by dairy cows in high-forage systems Animal Feed Science and Technology 2006 131 168 206 10.1016/j.anifeedsci.2006.04.016

[B45] Varela A Oliete B Moreno T Portela C Monserrrat L Carballo JA Sánchez L Effect of pasture finishing on the meat characteristics and intramuscular fatty acid profile of steers of the Rubia Gallega breed Meat Sci 2004 67 515 522 10.1016/j.meatsci.2003.12.005 22061527

[B46] Wood JD Richardson RI Nute GR Fisher AV Campo MM Kasapidou E Sheard PR Enser M Effects of fatty acids on meat quality: a review Meat Sci 2004 66 21 32 10.1016/S0309-1740(03)00022-6 22063928

[B47] Park RJ ALFORD Ratcliffe D Effect on meat flavor of period of feeding a protected lipid supplement to lambs J Food Science 1975 40 1217 1221 10.1111/j.1365-2621.1975.tb01055.x

[B48] Campo MM Nute GR Wood JD Elmore SJ Mottram DS Enser M Modelling the effect of fatty acids in odour development of cooked meat in vitro: part I—sensory perception Meat Sci 2003 63 367 375 10.1016/S0309-1740(02)00095-5 22062390

[B49] FAO/WHO/UNU Protein and amino acid requirements in human nutrition Report of a joint FAO/WHO/UNU expert consultation (WHO Technical Report Series) 2007 935 149 150 18330140

